# Localizing chronic Q fever: a challenging query

**DOI:** 10.1186/1471-2334-13-413

**Published:** 2013-09-03

**Authors:** Dennis G Barten, Corine E Delsing, Stephan P Keijmel, Tom Sprong, Janneke Timmermans, Wim JG Oyen, Marrigje H Nabuurs-Franssen, Chantal P Bleeker-Rovers

**Affiliations:** 1 Radboud Expertise Centre for Q fever, Department of Internal Medicine, Division of Infectious Diseases, Radboud University Nijmegen Medical Centre, Nijmegen, the Netherlands; 2 Nijmegen Institute for Infection, Inflammation and Immunity (N4i), Radboud University Nijmegen Medical Centre, Nijmegen, the Netherlands; 3 Department of Internal Medicine and Infectious Diseases, Canisius Wilhelmina Hospital, Nijmegen, the Netherlands; 4 Department of Medical Microbiology, Canisius Wilhelmina Hospital, Nijmegen, the Netherlands; 5 Department of Cardiology, Radboud University Nijmegen Medical Centre, Nijmegen, the Netherlands; 6 Department of Nuclear Medicine, Radboud University Nijmegen Medical Centre, Nijmegen, the Netherlands; 7 Department of Medical Microbiology, Radboud University Nijmegen Medical Centre, Nijmegen, the Netherlands; 8 Department of Internal Medicine/Division of Infectious Diseases 463, Radboud University Nijmegen Medical Centre, P.O. Box 9101, Nijmegen, HB, 6500, the Netherlands

**Keywords:** Chronic Q fever, *Coxiella burnetii*, ^18^F-fluorodeoxyglucose positron emission tomography, Echocardiography, Modified Duke criteria, Endocarditis

## Abstract

**Background:**

Chronic Q fever usually presents as endocarditis or endovascular infection. We investigated whether ^18^F-FDG PET/CT and echocardiography were able to detect the localization of infection. Also, the utility of the modified Duke criteria was assessed.

**Methods:**

Fifty-two patients, who had an IgG titre of ≥ 1024 against *C. burnetii* phase I ≥ 3 months after primary infection or a positive PCR ≥ 1 month after primary infection, were retrospectively included. Data on serology, the results of all imaging studies, possible risk factors for developing proven chronic Q fever and clinical outcome were recorded.

**Results:**

According to the *Dutch consensus on Q fever diagnostics*, 18 patients had proven chronic Q fever, 14 probable chronic Q fever, and 20 possible chronic Q fever. Of the patients with proven chronic Q fever, 22% were diagnosed with endocarditis, 17% with an infected vascular prosthesis, and 39% with a mycotic aneurysm. 56% of patients with proven chronic Q fever did not recall an episode of acute Q fever. Ten out of 13 ^18^F-FDG PET/CT-scans in patients with proven chronic Q fever localized the infection. TTE and TEE were helpful in only 6% and 50% of patients, respectively.

**Conclusions:**

If chronic Q fever is diagnosed, ^18^F-FDG PET/CT is a helpful imaging technique for localization of vascular infections due to chronic Q fever. Patients with proven chronic Q fever were diagnosed significantly more often with mycotic aneurysms than in previous case series. Definite endocarditis due to chronic Q fever was less frequently diagnosed in the current study. Chronic Q fever often occurs in patients without a known episode of acute Q fever, so clinical suspicion should remain high, especially in endemic regions.

## Background

Q fever is a zoonosis caused by *Coxiella burnetii*[1,2]. The acute form of Q fever is asymptomatic in 60% of patients. Patients with symptomatic disease usually present with mild flu-like symptoms, pneumonia or hepatitis [1,3]. Following primary infection, 1-5% of patients develop chronic Q fever [1,4-6]. In the literature, the most described localization of chronic Q fever is endocarditis, accounting for 60-80% of cases [1,2,7,8]. Less frequently reported manifestations of chronic Q fever include infections of aneurysms or vascular prostheses (9% of cases), chronic infections during pregnancy (5%) and other persistent infections (8%), such as osteomyelitis and chronic hepatitis [8,9]. However, following the recent Q fever epidemic in the Netherlands [10-12], substantially more patients have been diagnosed with an infected aneurysm or vascular prosthesis [4,13].

The diagnosis of chronic Q fever is challenging. Persistent infection usually develops insidiously and most patients present with non-specific symptoms such as low-grade fever, night sweats, weight loss, hepatosplenomegaly, and a persistently raised erythrocyte sedimentation rate (ESR) and C-reactive protein (CRP) [1,3,8,14,15]. Both serology and PCR aid the laboratory diagnosis of chronic Q fever [16,17]. High levels of antibodies to phase I more than 3 months after primary infection are found in chronic Q fever, whereas antibodies to phase II predominate after convalescence from acute Q fever without signs of chronic infection [5,16,18]. Localization of infectious foci is important, because, in addition to prolonged antimicrobial therapy, adjuvant therapeutic measures such as surgical drainage or graft replacement are often necessary [9,19]. This demonstrates the need for reliable imaging methods. Infected aneurysms or vascular prostheses can be identified by using computed tomography (CT) or ^18^F-fluorodeoxyglucose positron emission tomography (FDG-PET/CT) [20-23]. In case of Q fever endocarditis, however, the diagnosis is usually more complex and vegetations are rarely seen by echocardiography [18,24,25]. This commonly delays the diagnosis with several months [26].

From 2007 until 2010, the southern part of the Netherlands faced the largest outbreak of Q fever ever reported [4,10]. Physicians were confronted with an increasing number of patients with suspected chronic infection. The *Dutch Q fever consensus group* provided a new guideline on the diagnosis of chronic Q fever discriminating 3 categories: possible, probable and proven chronic Q fever [15]. We investigated whether FDG-PET/CT and echocardiography were able to detect the localization of infection in all patients with chronic Q fever treated at 2 hospitals specialized in Q fever in the Netherlands. In addition, the utility of the modified Duke criteria was assessed.

## Methods

### Study design and patients

All patients referred to Radboud University Nijmegen Medical Centre and Canisius Wilhelmina Hospital in Nijmegen, the Netherlands between August 2008 and March 2011 were retrospectively included if they fulfilled the following criteria: detection of *C. burnetii* DNA in serum or tissue by PCR ≥ 1 month after primary infection or an anti-phase 1 IgG titre of ≥ 1024 against *C. burnetii* phase I ≥ 3 months following acute Q fever. Patients without symptomatic acute infection were included if anti-phase I IgG remained > 1024 over the course of > 3 months, or if there was positive serum PCR over the course of > 1 month. The exclusion criterion was age < 18 years. For each patient a standardized case report form was completed. According to the Dutch law, this study was exempt from approval by an ethics committee, because of the retrospective character of this study and the anonymous storage of data.

### Diagnostic work-up

#### Serology and molecular detection

In 1994, the French National Centre for Rickettsial Diseases proposed a cut-off value for anti-phase I IgG of 1:800 for the diagnosis of chronic Q fever, using an in-house immunofluorescence assay (IFA) [16]. This cut-off value was adopted by the modified Duke criteria [27] and is considered as diagnostic for chronic Q fever in most literature. However, it is recently recognized that the results of Q fever IFA vary according to the centre in which they are carried out and the methods used (commercially available immunofluorescence kits) [28,29]. This also applies to the Dutch situation, where much higher anti-phase I IgG titres were measured, especially during the first months after acute infection [4]. The Dutch Q fever consensus group proposed a cut-off value for anti-phase I IgG of 1:1024 (immunofluorescence assay; Focus Diagnostics, Inc., Cypress, CA, USA), measured at least 3 months after acute infection, for the diagnosis of chronic Q fever in the Netherlands. In our study, sera were also tested for *C. burnetii* antibodies using a complement fixation test (CFT) (Institute Virion/Serion, GmbH, Würzburg, Germany), testing only anti-phase II antibodies.

#### Dutch consensus on chronic Q fever

The guideline on the classification of chronic Q fever [15], that has been developed by the *Dutch Q fever consensus group*, was used for diagnosis and classification of chronic Q fever in this study. This guideline uses a combined approach based on risk factors, symptoms, microbiological findings and imaging studies to discriminate 3 groups of chronic Q fever:

##### Proven chronic Q fever

Chronic Q fever is considered proven in case of (1) a positive *C. burnetii* PCR on blood or tissue without evidence for acute Q fever OR (2) IFA anti-phase I IgG ≥ 1024 is present > 3 months after acute infection AND definite endocarditis according to the modified Duke criteria OR (3) IFA ≥ 1024 for anti-phase I IgG AND proven vascular infection by abdominal ultrasound (AUS), CT, or FDG-PET/CT.

##### Probable chronic Q fever

Chronic Q fever is classified as probable when IFA anti-phase I IgG ≥ 1024 is present > 3 months after acute infection in combination with (1) valvular defects not meeting the modified Duke criteria OR (2) a known aneurysm and/or vascular or cardiac valve prosthesis without signs of infection by means of echocardiography, FDG-PET/CT, CT or AUS OR (3) suspected osteomyelitis or hepatitis as manifestation of chronic Q fever OR (4) pregnancy OR (5) symptoms of chronic infection OR (6) granulomatous tissue inflammation, histologically proven OR (7) being immunocompromised.

##### Possible chronic Q fever

Possible chronic Q fever is diagnosed when IFA anti-phase I IgG ≥ 1024 is present > 3 months after acute infection without manifestations meeting the criteria for proven or probable chronic Q fever.

#### Modified Duke criteria

The modified Duke criteria for infective endocarditis (IE) [27] were applied to all patients who underwent echocardiography. As a result, patients were stratified into 3 different groups: definite, possible and rejected IE. Besides the well-adopted modified Duke criteria by Li and colleagues [27], we also assessed 2 adjusted versions of these criteria that have been used previously in studies on Q fever endocarditis. In the first adjustment, the molecular (serum PCR) diagnosis of *C. burnetii* was considered as an additional major criterion [17,30]. In the second adjustment, the echocardiographic minor criteria that were eliminated by the modified Duke criteria in 2000 were reintroduced [27,31]. Echocardiographic minor criteria include nodular valvular thickening, nonoscillating targets, and new valvular fenestrations [31].

#### Imaging studies

Data on the following imaging studies were recorded: AUS, CT, FDG-PET/CT, transthoracic echocardiography (TTE) and transesophageal echocardiography (TEE). FDG-PET/CT scans were performed according to international guidelines [32], using integrated PET/CT scanners (Biograph™; Siemens, Knoxville, TN, USA or Gemini™, Philips, Eindhoven, the Netherlands). All FDG-PET/CT scans were performed in regular patient care and therefore reviewed by specialized nuclear radiologists from the department of Nuclear Medicine, Radboud University Nijmegen Medical Centre, Nijmegen, the Netherlands, as well as the department of Nuclear Medicine, Canisius Wilhelmina Hospital, Nijmegen, the Netherlands. Higher metabolic activity than physiological uptake in surrounding tissue in tissues with normally low physiological uptake was considered to be indicative of infection. In addition, irregular/localized FDG-uptake in tissues with normally homogenous uptake was considered indicative of infection. Each original report was used to score for relevant abnormal findings. If these findings enabled localization of infection, they were considered helpful. Abnormal results that gave rise to further analysis, i.e. suspected malignancy, but were not caused by chronic Q fever, were labelled as unexpected findings.

### Clinical data and outcome

Acute Q fever infection was regarded symptomatic if patients were diagnosed with Q fever pneumonia or if they could recall an episode of fever and pneumonia and/or headache, that was not caused by other known pathogens and that preceded the first positive Q fever serology or positive serum PCR. Patients were regarded to have pre-existing valvular disease if they were previously known with a valvulopathy ≥ grade II (stenosis or insufficiency, including congenital heart disease), or if they had a medical history of valve replacement. Valvular dysfunction was defined as the aggravation of pre-existing valvulopathies to ≥ grade 2, the occurrence of new valvulopathies of ≥ grade 2 or signs of artificial valve dysfunction, or evidence of increasing heart failure or the need for acute cardiac valve replacement. Data on other possible risk factors for chronic Q fever were collected (age, smoking, known aneurysm, presence of a vascular prosthesis, immunosuppression or –deficiency, other co-morbidity, and symptomatic acute Q fever). The diagnostic workup was considered complete if both echocardiography and screening for abdominal infection were completed. Patients were considered to be cured if their anti-phase I IgG antibody titre at least showed a fourfold decrease or had declined to < 1024 during subsequent serological testing, serum PCR had become and/or remained negative, and diagnostic imaging during follow-up showed no signs of active infection.

### Statistical methods

All data were analyzed using SPSS (version 16.0, SPSS, Inc.). Two-tailed Pearson’s chi-square tests or Fischer’s exact tests were used to compare qualitative data, whereas mean values were analyzed by Student’s t-tests. Differences were considered to be statistically significant at a p-value less than 0.05.

## Results

All 52 patients fulfilling the inclusion criteria were included in the study (Tables 1, 2, 3).

**Table 1:** Population characteristics of 52 patients with possible, probable and proven chronic Q fever*

	**Proven chronic Q fever:**	**Probable chronic Q fever:**	**Possible chronic Q fever:**
	**Number of patients**	**Number of patients**	**Number of patients**
	**(% or range)**	**(% or range)**	**(% or range)**
**General**			
Number of patients	18	14	20
Male sex	17 (94%)	8 (57%)	11 (55%)
Age at diagnosis	61 ± 16 yrs (26-88)	63 ± 12 yrs (43-84)	54 ± 15 yrs (26 – 81)
Mean BMI	25 ± 3 kg/m^2^ (18-30)	25 ± 4 kg/m^2^ (18-30)	25 ± 7 kg/m^2^ (19-41)
History of smoking	14 (78%)	9 (64%)	10 (50%)
Symptomatic acute infection	8 (44%)	12 (86%)	13 (65%)
Symptomatic chronic infection	14 (78%)	2 (14%)	0
Mean interval acute Q fever to analysis	12 ± 9 months (1-27)	16 ± 11 months (1-41)	7 ± 5 months (1-15)
Antibiotic therapy for chronic Q fever	18 (100%)	7 (50%)	3 (15%)
**Localization of infection**	**13 (72%)**	**2 (14%)**	**0**
Definite endocarditis	4 (22%)^†‡^	2 (14%)^§^	0
Vascular prosthesis	3 (17%)^‡^	0	0
Mycotic aneurysm	7 (39%)	0	0
Focus unknown	5 (28%)	12 (86%)	20 (100%)

^*^Adapted from Wegdam-Blans et al. [15].

^†^Definite endocarditis according to the modified Duke criteria.

^‡^One patient had a definite endocarditis according to the modified Duke criteria and an infected vascular prosthesis.

^§^Possible endocarditis according to the modified Duke criteria.

Bold texts summarize components of the table.

Abbreviation: *BMI* body mass index.

**Table 2:** Risk factors for developing chronic Q fever in 52 patients with possible, probable and proven chronic Q fever*

	**Proven chronic Q fever:**	**Probable chronic Q fever:**	**Possible chronic Q fever:**
	**Number of patients**	**Number of patients**	**Number of patients**
	**(% or range)**	**(% or range)**	**(% or range)**
Number of patients	18	14	20
**Pre-existing valvular disease**^**†**‡^	**5 (28%)**	**4 (29%)**	**0**
Mitral regurgitation	0	2 (14%)	0
Tricuspid regurgitation	0	1 (7%)	0
Bicuspid aortic valve	0	1 (7%)	0
Congenital (not bicuspid aortic valve)	1 (6%)	1 (7%)	0
Rheumatic fever	1 (6%)	0	0
Cardiac valve prosthesis^**†**^	4 (22%)	0	0
*Biological aortic prosthesis*	*3 (17%)*	*0*	*0*
*Biological mitral prosthesis*	*1 (6%)*	*0*	*0*
*Mechanical aortic prosthesis*	*1 (6%)*	*0*	*0*
**Known aneurysm**	**8 (44%)**	**1 (7%)**	**0**
Abdominal aortic aneurysm	7 (39%)	0	0
Dilated aortic root	1 (6%)	0	0
Cerebral aneurysm	0	1 (7%)	0
**Vascular prosthesis**	**11 (61%)**	**4 (29%)**	**0**
Abdominal aortic graft	7 (39%)	1 (7%)	0
Thoracic aortic graft	2 (11%)	0	0
PTA, iliacal or renal arteries	1 (6%)	2 (14%)	0
Goretex vascular shunt	1 (6%)	0	0
Coiling of cerebral aneurysm	0	1 (7%)	0
**Immunocompromised**	**1 (6%)**	**6 (43%)**	**0**
Immunosuppressive therapy	1 (6%)	4 (29%)	0
Myelodysplastic syndrome	0	2 (14%)	0
**Co-morbidity**^**†**^	**18 (100%)**	**14 (100%)**	**8 (40%)**
Chronic renal insufficiency	6 (33%)	4 (29%)	1 (5%)
Diabetes	3 (17%)	2 (14%)	4 (20%)
Active malignancy	1 (6%)	4 (29%)	1 (5%)
Systemic sclerosis	1 (6%)	2 (14%)	0
COPD	2 (11%)	3 (21%)	5 (25%)
Other^§^	5 (28%)	3 (21%)	3 (15%)

^*^Adapted from Wegdam-Blans et al. [15].

^†^Multiple predisposing conditions are possible for a patient.

^‡^Including cardiac valve prosthesis; valvulopathies were considered clinically significant if ≥ grade II.

^§^Including severe peripheral arterial disease, coronary artery bypass graft, congestive heart failure and liver cirrhosis.

Bold texts summarize components of the table.

Abbreviations: *PTA* Percutaneous transluminal angioplasty, *COPD* Chronic obstructive pulmonary disease.

**Table 3:** Diagnostics, treatment and outcomes in 52 patients with possible, probable and proven chronic Q fever*

	**Proven chronic Q fever:**	**Probable chronic Q fever:**	**Possible chronic Q fever:**
	**Number of patients**	**Number of patients**	**Number of patients**
	**(% or range)**	**(% or range)**	**(% or range)**
Number of patients	18	14	20
Serum PCR	12 (67%)	0	0
Tissue PCR	6 (33%)	0	0
Anti-phase I IgG at diagnosis	4096 (256-65536)	2048 (1024-32768)	2048 (1024-16384)
CFT at diagnosis	1280 (0-20480)	320 (80-5120)	320 (40-2560)
Time to anti-phase I IgG <1024 (months)	23.3 ± 7.9 [n = 4]	12.6 ± 3.9 [n = 5]	7.5 ± 5.1 [n = 8]
Time to negative serum PCR	3.6 ± 3.0 [n = 7]	NA	NA
Complete diagnostic workup	16 (89%)	9 (64%)	8 (40%)
**Abdominal ultrasound**	**8 (44%)**	**6 (43%)**	**8 (40%)**
Fluid collection	3	0	0
Increased diameter of aneurysm	1	0	0
Helpfulness	4/8 (50%)	0	0
**Screening abdominal CT**	**2 (11%)**	**1 (7%)**	-
Aneurysm	2	0	-
Suggestive of infected aneurysm or prosthesis	1	0	-
Helpfulness	2/2 (100%)	0	-
**CT on account of PET/CT**	**3 (17%)**	**0**	**0**
Aneurysm	2	-	-
Suggestive of infected aneurysm or prosthesis	3	-	-
Helpfulness	3/3 (100%)	-	-
**FDG-PET/CT**	**13 (72%)**	**8 (57%)**	**9 (45%)**
Focal uptake aneurysm	7	0	0
Focal uptake vascular prosthesis	3	0	0
Soft tissue inflammation	4	0	0
Para-aortal lymfadenopathy	1	0	0
Mediastinal lymfadenopathy	1	3	0
Unexpected findings	4	4	2
Helpfulness	10/13 (77%)	0	0
**TTE**	**16 (89%)**	**13 (93%)**	**12 (60%)**
Echocardiographic major criteria	0	0	0
Echocardiographic minor criteria	12	8	4
Helpfulness	1/16 (6%)	1/13^†^ (8%)	0
**TEE**	**6 (33%)**	**3 (21%)**	**4 (20%)**
Echocardiographic major criteria	2	0	0
Echocardiographic minor criteria	6	1	3
Helpfulness	3/6 (50%)	1/3^†^ (33%)	0
**Antibiotic therapy**	**18 (100%)**	**7 (50%)**	**3 (15%)**
Mortality during treatment	3	0	0
Ongoing treatment	13	7	2
Treatment completed successfully	2	0	1
Mean duration of treatment (months)	21.5 ± 6.4 [n = 2]	-	2 [n = 1]
**Surgery**	**6 (33%)**	**0**	**0**
Aortic graft surgery^‡^	4 (22%)	-	-
Cardiac valve surgery	2 (11%)	-	-
**Mortality**	**3 (17%)**	**0**	**0**

^*^Adapted from Wegdam-Blans et al. [15].

^†^TTE and TEE were considered helpful in 2 patients where pre-existing valvulopathies aggravated.

^‡^One patient had surgery twice.

Bold texts summarize components of the table.

Abbrevations: *PCR* Polymerase chain reaction, *CFT* Complement fixation test, *NA* Not applicable, *CT* Computed tomography, ^*18*^*F DG-PET/CT*^18^F- fluorodeoxyglucose positron emission tomography combined with CT, *TTE* Transthoracic echocardiography, *TEE* Transesophageal echocardiography.

### Proven chronic Q fever

Proven chronic Q fever was diagnosed in 18 patients (Table 1). One patient developed systemic sclerosis during treatment. Only 8 patients (44%) recalled an episode of acute Q fever. Fourteen patients (78%) had symptomatic chronic infection: fever (9/14), abdominal pain (4/14), fatigue (3/14), weight loss (3/14), valvular dysfunction (3/14), night sweats (2/14) or lumbar pain (2/14). In two out of five patients with a pre-existing valvulopathy, valvular dysfunction occurred (left ventricular function deterioration due to Q fever endocarditis, and a new dysfunction of an artificial cardiac valve, as a consequence of Q fever endocarditis). One patient with valvular dysfunction was not familiar with a previous valvulopathy. The mean interval between symptomatic acute Q fever and the diagnosis of chronic Q fever was 12 ± 9 months (range: 1-27). Definite endocarditis was diagnosed in 4 patients (22%), an infected vascular prosthesis in 3 patients (17%), and an infected aneurysm in 7 patients (39%). One of these patient had both a definite endocarditis and an infected vascular prosthesis. In 5 patients (28%), no definite focus was identified. According to the modified Duke criteria, 4 of these patients had possible endocarditis and the fifth patient declined further diagnostic tests due to his age and underlying medical condition.

**Figure 1:**
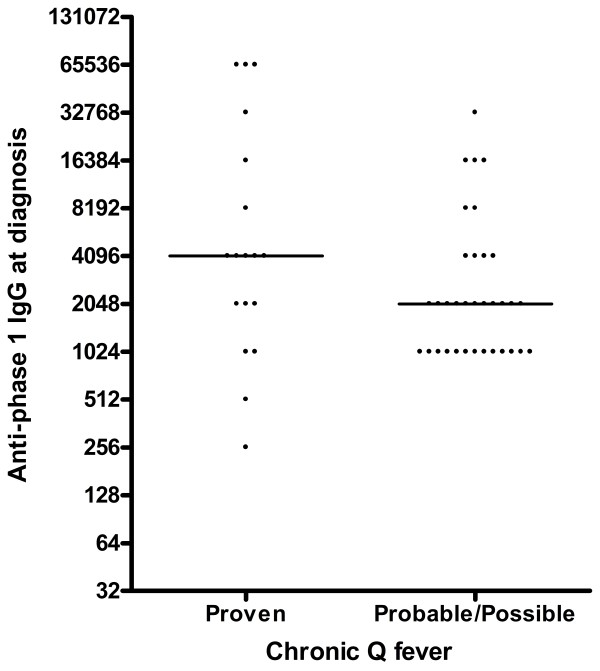
Titres of anti-phase 1 IgG at the time of chronic Q fever diagnosis.

**Figure 2:**
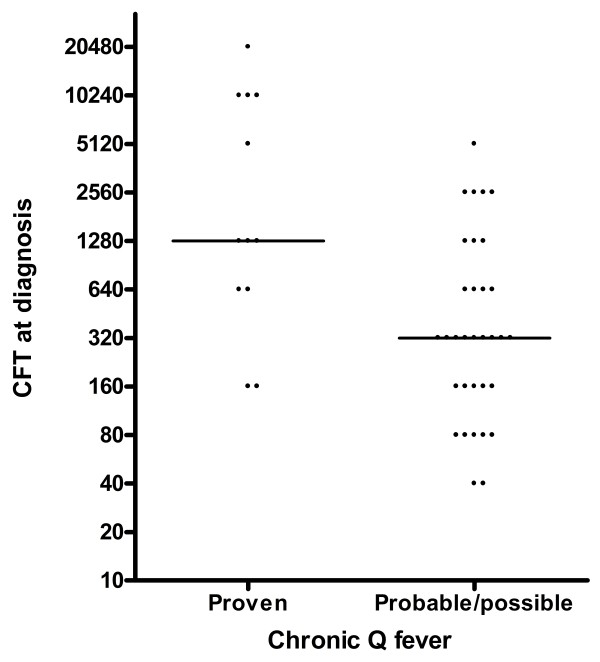
**Titres of complement fixation test at the time of chronic Q fever diagnosis.** Abbrevations: *CFT* Complement fixation test.

The median anti-phase I IgG titre at diagnosis was 4096 (range: 256-65536), and the median height of CFT was 1280 (range: 0-20480) (Figures 1 and 2). One patient had an anti-phase I IgG titre of only 256 and a negative CFT at diagnosis, but was considered to have proven chronic Q fever because serum PCR tested positive > 1 month following primary infection. In 4 patients, the anti-phase I IgG titre decreased to < 1024 after a mean duration of treatment of 23.3 ± 7.9 months. By PCR, *C. burnetii* DNA was successfully isolated from tissue samples (cardiac valve, vascular prosthesis) in 5 out of 6 patients who underwent surgery (1 patient underwent surgery twice). There was 1 positive PCR on fluid spontaneously draining from a fistula between an abscess around a vascular prosthesis and the skin. Four out of the 6 patients with positive fluid/tissue PCR were analyzed by FDG-PET/CT, all of which showed FDG-positive lesions. The other 2 were already found to have definite IE according to the modified Duke criteria and no FDG-PET/CT was performed. In these 2 patients, PCR was positive on infected cardiac valves that were replaced by surgery. In 7 of 12 patients with a positive serum PCR, PCR became negative after an average of 3.6 ± 3.0 months. Two patients died when PCR had not become negative yet, 1 patient was lost to follow-up, and 2 patients still had a positive serum PCR after 4 and 6 weeks of treatment, respectively.

**Figure 3:**
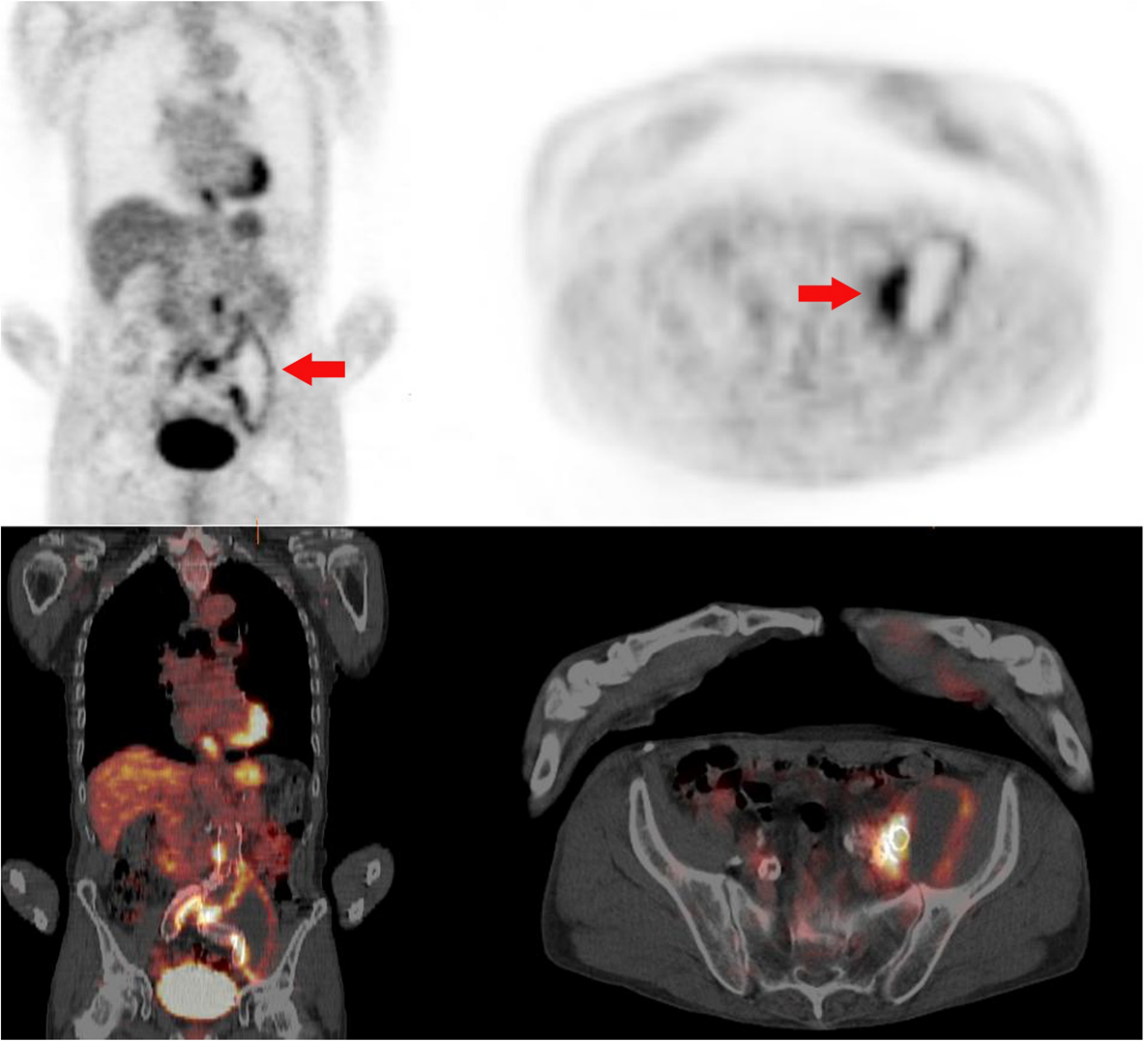
^**18**^**F-FDG PET/CT image demonstrating a mycotic aneurysm. **^18^F-FDG PET/CT images (left column coronal sections, right column transverse sections, upper row PET images, lower row PET/CT fusion images) of a patient with proven chronic Q fever demonstrating a mycotic aneurysm and associated abscess adjacent to the left common iliac artery (arrows). Abbrevations: ^*18*^*F DG-PET/CT*^18^F- fluorodeoxyglucose positron emission tomography combined with CT.

A complete diagnostic work-up for chronic Q fever was performed in 16 patients (89%) (Table 3). In 2 patients, this work-up was incomplete: 1 patient refused further analysis, and in 1 patient only echocardiography was done. In 13 patients (72%), FDG-PET/CT was performed, which was helpful in identifying the site of infection in 10 of 13 investigations (77%). All 7 patients with an aneurysm as identified site of Q fever infection showed focal FDG-uptake of the aneurysm. Furthermore, all 3 patients with a vascular prosthesis as identified site of Q fever infection showed focal uptake around the vascular prosthesis (Figure 3). In 4 out of the 13 above mentioned FDG-PET/CT scans, FDG-positive lesions were confirmed by positive *C. burnetii* PCR on tissue. In all of these 4 patients, FDG-PET/CT was conducted prior to PCR. In the remaining patients, surgery was not indicated and the lesions were very difficult to reach so tissue PCR could not be performed. Unexpected findings were observed in 4 patients (31%). As a result, 2 patients required a biopsy because of focal FDG-uptake in the lungs, leading to the diagnosis of lung carcinoma in 1 patient and fibrosis in the other. In 1 patient, massive mediastinal lymphadenopathy was seen, eventually leading to the diagnosis of systemic sclerosis. CT was performed in 5 patients. Two of these investigations were done initially (‘screening abdominal CT’), and the remaining 3 were conducted on account of a preceding abnormal FDG-PET/CT-scan (one chest CT and two CT scans of both chest and abdomen). Both screening CT-scans enabled localization of infection and were considered helpful. The 3 CT-scans that were performed on the basis of pathology on FDG-PET/CT all confirmed the abnormal FDG-PET/CT findings. TTE was performed in 16 patients (89%); none of these examinations showed a major criterion, whereas echocardiographic minor criteria were seen in 12 patients (75%). Nevertheless, TTE was regarded helpful in 1 patient where nodular valvular thickening of an aortic bioprosthesis was seen. TEE was performed in 6 patients (33%), 4 following a prior TTE. In 2 patients, an echocardiographic major criterion was observed, whereas echocardiographic minor criteria were recorded in all of the performed TEEs. In 3 patients (50%), TEE was considered helpful: 2 because of echocardiographic major criteria and 1 as a result of aggravated pre-existing valvular disease. In 4 out of 5 patients with no definite localization and possible IE there were minor echocardiographic criteria. In all 5 patients TTE was performed. Two out of 5 TTE’s showed minor criteria. In 4 patients TEE was performed, 3 of which showed minor criteria.

Long-term antibiotic treatment (doxycycline 200 mg/day and hydroxychloroquine 600 mg/day) was given to all patients. Thirteen patients (72%) are still under treatment, 3 of whom are being treated for more than 18 months. Three patients (17%) died during the course of therapy as a consequence of chronic Q fever infection. Death from chronic Q fever was defined as death as a result of active chronic infection. One patient died at 11 months following cardiac valve replacement due to progressive heart failure, probably as a result of artificial valve dysfunction due to chronic Q fever. PCR on valve tissue was positive. The second patient died in the perioperative period (in the first month) due to bleeding following acute aneurysm repair for a symptomatic aneurysm. PCR on aneurysm tissue was positive for Q fever. The third patient died in the perioperative period (in the first month) due to SIRS following acute cardiac valve replacement for severe Q fever endocarditis, with tissue PCR being positive. In 2 patients (11%), treatment was completed successfully after a treatment duration of 18 and 26 months with a follow-up after completion of treatment of 16 and 4 months, respectively. Six patients (33%) underwent surgery: abdominal aortic graft surgery with open repair was performed in 1 patient, endovascular aneurysm repair (EVAR) in 2 patients, first EVAR later followed by abscess drainage, excision of infected tissue and lavage with omentum plasty in 1 patient, and cardiac valve replacement in 2 patients.

### Probable chronic Q fever

Probable chronic Q fever was diagnosed in 14 patients (Table 1). In this group, 6 patients (43%) were immunocompromised (Table 2). Twelve patients (86%) experienced symptomatic acute infection in the past. Two patients (14%) experienced symptoms of chronic infection: fever and night sweats (n = 1), and weight loss and fatigue (n = 1). The mean interval between acute Q fever and analysis for chronic infection was 16 ± 11 months (range: 1-41 months). While in 12 patients (86%) no focus was localized, endocarditis (possible endocarditis according to the modified Duke criteria) was regarded as the most probable site of infection in 2 cases.

The median anti-phase I IgG titre at first analysis was 2048 (range: 1024-32768) and the median CFT-value was 320 (range: 80-5120). In 5 patients the anti-phase I IgG titre decreased to < 1024. Of these 5 patients, 2 received treatment for chronic Q fever and their anti-phase I IgG titre decreased to < 1024 in 12 and 18 months, respectively. In the 3 patients without treatment, anti-phase I IgG titre decreased to < 1024 after 4, 10, and 12 months, respectively.

A complete diagnostic work-up was performed in 9 patients (64%) (Table 3). Two patients were asymptomatic and considered low-risk, 1 patient refused further analysis because of co-morbidity, and in 2 patients FDG-PET/CT was postponed (because of recent surgery and a concomitant severe pneumonia, respectively). FDG-PET/CT was performed in 8 patients (57%). None of these investigations localized infection (otherwise the patient would have proven chronic Q fever). Four of the performed FDG-PET/CT-scans (50%) revealed unexpected findings. These include mediastinal lymphadenopathy (eventually leading to the diagnosis of systemic sclerosis) in 1 patient, and focal FDG-uptake in the dental region in another patient. In 1 patient with multiple enlarged mediastinal lymph nodes, subsequent broncheoalveolar lavage (BAL) did not lead to a definitive diagnosis. In 1 patient, multiple unexpected findings were observed (focal uptake in the left thyroid gland followed by hemithyroidectomy leading to a diagnosis of adenoma and multiple foci in the prostate and iliac bone, leading to the diagnosis of prostate carcinoma). TTE was performed in 13 patients (93%) and was considered helpful once (8%), because progression of pre-existing valvular disease was observed. In 8 patients (62%), echocardiographic minor criteria were recorded. TEE was performed in 3 patients (21%), which was helpful in 1 patient. Echocardiographic minor criteria were seen in 1 patient (33%).

Seven patients (50%) received long-term treatment with antibiotics (doxycycline 200 mg/day and hydroxychloroquine 600 mg/day); none of these patients completed treatment yet. Of the remaining 7 patients, a decision on treatment was pending in 2 patients, 3 were not treated because of severe co-morbidity, and 3 were asymptomatic and considered low-risk. All patients that were not on antibiotic treatment were followed closely.

### Possible chronic Q fever

Twenty patients were diagnosed with possible chronic Q fever (Table 1). Thirteen (65%) patients could recall a symptomatic episode of acute Q fever and none of the patients experienced symptoms of chronic infection. The mean interval between acute infection and analysis for chronic Q fever was 7 ± 5 months (range: 1-15 months). In 77% of the patients with possible chronic Q fever and a previously known episode of acute Q fever, routine serological follow-up at 3, 6, 9 and 12 months was performed.

The median anti-phase I IgG titre at first analysis was 2048 (range: 1024-16384), and the median CFT-value was 320 (range: 40-2560). In 8 patients, the anti-phase I IgG titre decreased to < 1024, with an average of 7.5 ± 5.1 months. Of these patients, 1 patient was treated and the anti-phase I IgG titre decreased to < 1024 within 7 months.

A complete diagnostic work-up was performed in 8 out of 20 patients (40%) (Table 3). Six patients were asymptomatic and considered low-risk, 1 patient suffered from severe co-morbidity, 3 patients were lost to follow-up, and 2 patients were not yet completely analyzed. FDG-PET/CT was performed in 9 patients (45%). None of these investigations were helpful. Two FDG-PET/CT-scans (22%) resulted in an unexpected finding: 1 patient with FDG-uptake in the colon, followed by colonoscopy diagnosing a non-neoplastic polyp, and 1 patient with FDG-uptake in the left clavicle, followed by CT that was normal. TTE was performed in 12 patients (60%) and was considered helpful in none of the investigations. In 4 patients (33%), echocardiographic minor criteria were recorded. TEE was performed in 4 patients (20%), being helpful in none of the patients. Echocardiographic minor criteria were seen in 3 patients (75%).

Long-term antibiotic treatment was prescribed to three patients (15%) because of debilitating symptoms (severe fatigue and muscle ache). One of these patients initially started treatment because of suspected chronic Q fever, but stopped after 2 months because anti-phase I IgG titres were rapidly decreasing. The 2 other patients had not completed treatment yet. Of the remaining 17 patients, 11 were considered low-risk, in 5 a decision on treatment was pending, and 1 patient had severe co-morbidity.

### Comparison between patients with proven chronic Q fever and patients with probable and possible chronic Q fever

In order to evaluate potential differences between patients with proven chronic Q fever and those with possible or probable chronic Q fever, data were compared by univariate analysis (Table 4). Age at diagnosis, history of smoking, and mean interval from acute infection to analysis for chronic Q fever did not differ significantly between the groups. Male sex (p = 0.04) and symptomatic chronic infection (p < 0.01) were significantly more present in patients with proven chronic Q fever. Concerning risk factors, which were found previously in other studies, the presence of pre-existing valvular disease, indication for endocarditis prophylaxis, and immunodeficiency did not differ significantly between the groups in our study. In contrast, cardiac valve prostheses (p = 0.01), known aneurysms (p < 0.01), and vascular prostheses (p < 0.01) were significantly associated with proven chronic Q fever.

**Table 4:** Significant differences between patients with proven chronic Q fever and patients with probable and possible chronic Q fever (univariate analysis)*

	**Proven chronic Q fever:**	**Probable and possible chronic Q fever:**	**Significance (p)**
	**Number of patients**	**Number of patients**	
	**(% or range)**	**(% or range)**	
**Patient characteristics**			
Number of patients	18	34	
Male sex	17 (94%)	19 (56%)	0.04
Symptomatic chronic infection	14 (78%)	2 (6%)	<0.0001
Cardiac valve prosthesis	4 (22%)	0	0.01
Known aneurysm	8 (44%)	1 (3%)	0.0004
Abdominal aortic aneurysm, infrarenal	7 (39%)	0	0.0003
Vascular prosthesis	11 (61%)	4 (12%)	0.004
Co-morbidities	18 (100%)	22 (65%)	0.021
**Diagnostic work-up**	**16 (89%)**	**17 (50%)**	**0.04**
Positive serum PCR	12 (67%)	0	<0.0001
Positive tissue PCR	6 (33%)	0	0.011
Anti-phase I IgG at diagnosis	4096 (256-65536)	2048 (1024-32768)	0.013
CFT at diagnosis	1280 (0-20480)	320 (40-5120)	0.001
Time to anti-phase I IgG <1024 (months)	23.3 ± 7.9 [n = 4]	9.5 ± 5.2 [n = 13]	0.001
Helpfulness of abdominal ultrasound	4/8 (50%)	0/14 (0%)	0.01
Helpfulness of FDG-PET/CT	10/13 (77%)	0	<0.0001
**Antibiotic therapy**	**18 (100%)**	**10 (29%)**	**<0.0001**
Mortality during treatment	3/18 (17%)	0	0.037
Ongoing treatment	13/18 (72%)	9/10 (90%)	0.008
**Surgery**	**6 (33%)**	**0**	**0.001**
Aortic graft surgery^†^	4 (22%)	0	0.011
Cardiac valve surgery	2 (11%)	0	NS
**Mortality**	**3 (17%)**	**0**	**0.033**

^*^Adapted from Wegdam-Blans et al. [15].

^†^One patient had surgery twice.

Bold texts summarize components of the table.

Abbrevations: *PCR* polymerase chain reaction, *CFT* complement fixation test, *FDG-PET/CT*^18^F- fluorodeoxyglucose positron emission tomography, *NS* not significant.

Anti-phase I IgG (p = 0.01) and CFT-values (p < 0.01) were significantly higher in patients with proven chronic Q fever when compared to the groups of probable and possible Q fever combined (Figures 1 and 2). Also, the mean time to anti-phase I IgG < 1024 was significantly longer in this group (p < 0.01). In contrast to AUS and FDG-PET/CT, the helpfulness of CT, TTE and TEE showed no significant differences between the groups. Both antibiotic treatment (p < 0.01) and surgery (p < 0.01) were used more often in patients with proven chronic Q fever. Most importantly, a clear association was seen between proven chronic Q fever and mortality rates (p = 0.03).

### Analysis after adjustments to the modified Duke criteria

The modified Duke criteria and the 2 aforementioned adjustments to these criteria were applied to all patients (Table 5). Applying the modified Duke criteria, 4 cases of definite IE were diagnosed, and 20 cases of possible IE. Of 20 patients with possible IE (all groups), 11 out of 19 patients who underwent TTE had minor criteria by TTE, and 4 out of 7 patients who underwent TEE had minor criteria. When echocardiographic minor criteria were included (first adjustment), 8 cases were considered definite IE and 28 cases possible IE. Including a positive serum PCR for *C. burnetii* as a major criterion (second adjustment), 12 patients scored definite IE and 14 possible IE. The modified Duke criteria were compared with the modified Duke criteria including our first and second adjustments, respectively, by a 2-tailed Wilcoxon-test, which showed significant differences (p = 0.046 and p < 0.01, respectively).

**Table 5:** Comparison of (adjustments to) modified Duke criteria: complete case series

	**Modified Duke criteria **[27]	**Modified Duke criteria, including echocardiographic minor criteria **[31]	**Significance**^**† **^**(comparison with modified Duke criteria) (p)**^**‡**^	**Modified Duke criteria, including PCR as a major criterion **[30]	**Significance**^**† **^**(comparison with modified Duke criteria) (p)**^**§**^
**Definite IE**	4 (9%)	8 (19%)	0.046	12 (28%)	0.005
**Possible IE**	20 (47%)	28 (65%)	0.046	14 (33%)	0.034
**Rejected IE**	19 (44%)	7 (16%)	0.001	17 (40%)	0.157
**Total**	43^*^	43^*^	-	43^*^	-

^†^Wilcoxon test, 2-tailed.

^‡^Modified Duke criteria compared to ‘modified Duke criteria, including echocardiographic minor criteria’.

^§^Modified Duke criteria compared to ‘modified Duke criteria, including PCR as a major criterion’.

^*^Nine patients were not examined by echocardiography; the modified Duke criteria could therefore not be calculated.

Abbrevations: *PCR* polymerase chain reaction, *IE* infective endocarditis.

## Discussion

In this study, the diagnostic work-up of 52 patients with chronic Q fever according to the *Dutch consensus on Q fever diagnostics* was evaluated. We demonstrated that FDG-PET/CT might be a valuable tool for localization of vascular infection with *C. burnetii*. It was shown that infected aneurysms or vascular prostheses are the most common manifestation of proven chronic Q fever in our population.

The mean age of patients was similar to previously reported case series of chronic Q fever [6,8,17]. The overall male predominance has been shown before as well, but the portion of male patients with proven chronic Q fever (94%) was distinct. This possibly results from a higher incidence of aneurysms and cardiovascular disease in male subjects, which are clear risk factors for developing chronic Q fever [3,8,33]. A history of smoking was established as a risk factor for chronic Q fever, especially in those patients with proven chronic Q fever. Smoking was not included in the possible risk factors for developing chronic Q fever in the recently published Dutch study by Kampschreur et al. [33]. Furthermore, patients with proven chronic Q fever more often had a cardiac valve prosthesis, a known aneurysm, or a vascular prosthesis as was also found by Kampschreur et al. [33]. Although reported in some previous studies, pre-existing valvular disease other than valve prosthesis did not appear to be an important risk factor in this study [6,14,33]. A similar observation was done by another Dutch group [34,35], that found a low risk of progression to Q fever endocarditis in the presence of degenerative valvular disease.

Only 44% of patients with proven chronic Q fever could recall an episode of acute Q fever, compared to 74% of those with possible/probable Q fever. Symptomatic acute infection most often results in antibiotic treatment, which might reduce the chance of developing proven chronic Q fever. In addition, in patients with acute Q fever, serological follow-up is performed while this was of course not the case in patients without symptomatic (and thus usually unknown) infection. It is possible that elevating titres of IgG anti-phase I titres found during follow-up led to earlier diagnosis and treatment, possibly preventing progression from possible and probable chronic Q fever to proven chronic Q fever.

A large retrospective study from France identified endocarditis as the predominant manifestation of chronic Q fever (73% of cases) [8]. In contrast, only 22% of our patients with proven chronic Q fever have been diagnosed with Q fever endocarditis. Infected aneurysms and infected vascular prostheses were found in 39% and 17% of patients, respectively. It has been suggested that mycotic aneurysms may be caused by non-diagnosed endocarditis in patients with chronic Q fever. However, applying the modified Duke criteria to all patients with proven chronic Q fever, only 1 patient had an infected vascular prosthesis and definite IE at the same time. One patient with an infected vascular prosthesis and 1 patient with an infected aneurysm had rejected IE according to the modified Duke criteria. The last patient with an infected vascular prosthesis and all other patients with an infected aneurysm had possible IE according to the modified Duke criteria. The cause of this striking difference in predominant manifestation of chronic Q fever remains largely unclear, and probably results from a combination of factors. First, most patients in other series were evaluated because of endocarditis, whereas in our case series, also other complaints (fever, night sweats, presence of aneurysm) and routine serological follow up after acute Q fever led to evaluation for Q fever because of the current epidemic. In addition, not all patients underwent echocardiography, possibly leading to an underestimation of endocarditis in our group. Furthermore, it is possible that in those patients without a full diagnostic work-up only one site of infection was notified, whereas it is possible that patients had 2 sites of infection. Second, pre-existing valvular disease was seen less often in this case series than in those patients reported in literature. This could be influenced by the fact that screening echocardiography is not performed in patients with acute Q fever in the Netherlands. Although our study did not found pre-existing valvular disease to be a significant risk factor for proven chronic Q fever, this contrasts with previous studies [6,14], but is in accordance with the other Dutch study on risk factors for developing proven chronic Q fever [33]. Third, the Dutch *C. burnetii* strain is possibly more likely to cause endovascular infection other than endocarditis. Even though it is possible that more vascular infections were found because FDG-PET/CT was performed more often, it is unlikely that vascular infections would go unnoticed in other chronic Q fever series, in which hardly any vascular infection was seen. If these patients would have had unidentified vascular infection in addition to endocarditis, more complications would be expected because of the high mortality rate of vascular chronic Q fever, even in case of optimal (surgical) treatment. Finally, it is not clear if other research groups applied the modified Duke criteria in the same strict manner as we did for this study.

In 1994, Durack et al. [31] introduced a new set of diagnostic criteria for IE that subsequently came to be known as the Duke criteria. Li and colleagues [27] proposed modifications to the Duke criteria in 2000, adding a positive serology for *C. burnetii* as a major criterion, which had already been proposed earlier by Fournier et al. [18]. In addition, the modifications included the elimination of echocardiographic minor criteria, because a widespread use of TEE was assumed. It is well-recognized that the sensitivity of these criteria is diminished in Q fever endocarditis, since it is known for its subtle valve abnormalities and absence of vegetations [4,18,24]. Nonetheless, we strictly applied the modified Duke criteria to this case series, resulting in only 4 patients (9%) with definite IE and 20 patients (47%) with possible IE. Even if we merely reflect on patients with proven chronic Q fever, the percentage of definite IE was only 22%. There were another 5 patients (28%) with an unidentified focus, 4 of whom had possible IE according to the modified Duke criteria. TEE was performed in a minority of patients (25%), while the elimination of echocardiographic minor criteria was based on the widespread use of TEE [27]. We cannot rule out the possibility that in some patients vegetations were missed because TTE was conducted exclusively.

In the past, several adjustments have been proposed to further improve the sensitivity of the modified Duke criteria. One of these adjustments was the use of PCR techniques as a major criterion [30], which is not implemented in international guidelines. However, in a recent study on Q fever endocarditis [6], a positive serum PCR served as major criterion. It is not clear whether PCR was an additional major criterion or served as substitute for serology. The theoretical addition of a positive serum PCR as major criterion to the modified Duke criteria appeared most useful. From our experience, we suggest that a positive serum PCR for *C. burnetii* in patients with chronic Q fever without an identified site of infection should be treated as Q fever endocarditis. Furthermore, the presence of echocardiographic minor criteria should raise the clinician’s suspicion of endocarditis, and TEE should be performed in all patients with chronic Q fever with an unknown focus. It is essential to bear in mind that the Duke criteria are useful for the classification of IE, but that they were designed for research purposes and thus should not replace clinical judgment in clinical practice.

CT was performed initially in only 2 patients with proven chronic Q fever, making it impossible to estimate the helpfulness of this technique. In contrast, FDG-PET/CT, localized infection in 77% of patients with proven chronic Q fever, which suggests that FDG-PET/CT is a valuable tool for the localization of vascular Q fever infection. FDG-PET/CT is also very well suited for diagnosing osteomyelitis, which is another possible focus of chronic Q fever. A well-recognized disadvantage of FDG-PET/CT is its specificity, as it does not differentiate between inflammation, infection, and malignancy. As such, unexpected findings were observed in 9 patients (30%), including the detection of previously unknown malignancies in 2 patients and newly diagnosed systemic sclerosis in another 2 patients. Five patients underwent invasive diagnostic procedures as a result of suspected malignancies, but pathological examination remained negative. The number of unexpected findings is higher than found in previous studies on the use of FDG-PET in other infections and fever of unknown origin (FUO) [36,37], which might be explained by the higher age of the patients and the male predominance in combination with a higher than average percentage of smokers, increasing the risk of associated malignancy when compared to patients with FUO. A limitation of our study is of course its retrospective character. Unfortunately, not all patients underwent a complete diagnostic work-up. Therefore, it is important to bear in mind that some patients might have had two sites of infection, which might have been missed. This emphasizes the need for a full diagnostic work-up in patients with chronic Q fever. Also, the time point of diagnostic imaging in the course of infection differed between the patients, which might have influenced the helpfulness.

## Conclusions

In conclusion, if chronic Q fever is diagnosed, FDG-PET/CT is a helpful imaging technique for localization of vascular infection. Patients with proven chronic Q fever were diagnosed significantly more often with mycotic aneurysms than in previous case series. Theoretical adjustment of the modified Duke criteria by adding serum PCR as a major criterion results in more diagnoses of Q fever endocarditis. We recommend treating patients with chronic Q fever with a positive serum PCR for *C. burnetii* without an identified site of infection as Q fever endocarditis. To increase sensitivity after previous exclusion of echocardiographic minor criteria from the modified Duke criteria, TEE is recommended in patients with chronic Q fever. A minority of all patients with proven chronic Q fever recalls a previous episode of acute Q fever, so clinical suspicion should remain high, especially in endemic regions.

## Abbreviations

AUS: Abdominal ultrasound; BAL: Broncheoalveolar lavage; C. burnetii: *Coxiella burnetii*; CFT: Complement fixation test; CT: Computed tomography; CRP: C-reactive protein; ESR: Erythrocyte sedimentation rate; EVAR: Endovascular aneurysm repair; FDG-PET/CT: ^18^F-fluorodeoxyglucose positron emission tomography; FUO: Fever of unknown origin; IE: Infective endocarditis; IFA: Immunofluorescence assay; PCR: Polymerase chain reaction; TEE: Transesophageal echocardiography; TTE: Transthoracic echocardiography.

## Competing interests

The authors declare that they have no competing interests.

## Authors’ contributions

CB and CD planned and designed the research study, and have been involved in the analysis and interpretation of data, as well as critical revision of the manuscript. DB has been involved in the design and acquisition of data, has done the analysis and interpretation of data and drafted the manuscript. SK has been involved in the design of the study, and participated in interpretation of results and critical revision of the manuscript. JT and WO participated in interpretation of results and revision of the manuscript. TS and MN provided microbiological expertise and patient data. All authors read and approved the final manuscript.

## Pre-publication history

The pre-publication history for this paper can be accessed here:

http://www.biomedcentral.com/1471-2334/13/413/prepub
